# Comparison of manual and automated ultrasensitive assays for residual HIV-1 in plasma from individuals on suppressive antiretroviral therapy

**DOI:** 10.1128/jcm.00995-25

**Published:** 2025-11-18

**Authors:** Sonia Bakkour Coco, Mars Stone, Xutao Deng, Yunfei Wang, Wes Rountree, Salvatore R. Scianna, Leilani Montalvo, Melanie Dimapasoc, Martin Stengelin, George Sigal, Guoxin Wu, Bonnie J. Howell, Sarah Palmer, Cheryl Jennings, Douglas D. Richman, Robert J. Gorelick, Gregory M. Laird, Albine Martin, Jana L. Jacobs, John W. Mellors, Steven G. Deeks, Michael P. Busch

**Affiliations:** 1 Vitalant Research Institute 166672, San Francisco, California, USA; 2 Department of Laboratory Medicine, University of California San Francisco 8785, San Francisco, California, USA; 3 Duke Human Vaccine Institute, Duke University Medical Center 609772, Durham, North Carolina, USA; 4 Meso Scale Diagnostics, LLC. 60915 , Rockville, Maryland, USA; 5 Merck & Co., Inc. 2793 , Rahway, New Jersey, USA; 6 The Westmead Institute for Medical Research, The University of Sydney 4334, Sydney, New South Wales, Australia; 7 Rush University Medical Center 2468, Chicago, Illinois, USA; 8 University of California San Diego 8784, La Jolla, California, USA; 9 AIDS and Cancer Virus Program, Frederick National Laboratory for Cancer Research 437329 , Frederick, Maryland, USA; 10 Accelevir Diagnostics, Baltimore, Maryland, USA; 11 Department of Medicine, University of Pittsburgh 6614, Pittsburgh, Pennsylvania, USA; 12 Department of Medicine, Division of HIV, Infectious Diseases & Global Medicine, University of California San Francisco 8785, San Francisco, California, USA; St Jude Children's Research Hospital, Memphis, Tennessee, USA

**Keywords:** antiretroviral therapy, HIV persistence, residual HIV viremia, HIV

## Abstract

Several nucleic acid and antigen assays have been developed to detect HIV in plasma at levels below the detection limit of standard clinical assays; however, comparative assessment of performance of these ultrasensitive assays on identical panels has been limited. Here, we report the relative performance characteristics of three manual (ultracentrifugation concentration and laboratory-developed PCR) and two automated commercial assays for single-copy detection of HIV RNA, as well as two ultrasensitive HIV p24 assays, using blinded panels of plasma samples containing HIV at low concentrations. Independent sample sets were studied in two phases: in the first phase, qualification panels consisted of analytic standards in serial dilution and clinical plasma samples from virologically suppressed people with HIV (PWH). HIV detection in clinical samples was infrequent using the ultrasensitive p24 assays (mean 11%). In contrast, a higher proportion of the same samples were detected using single-copy RNA assays (mean 61%). In the second phase, evaluation panels of clinical plasma samples (*n* = 80) from virally suppressed PWH and low-copy analytic standards assessed performance of one automated and two manual single-copy RNA assays with the highest qualification phase detection frequencies. The automated assay performed comparably in three separate laboratories and consistently detected HIV RNA in over half (mean 57%) of samples from virally suppressed PWH, whereas the manual assays detected HIV in ≤40% of the same samples. An automated single-copy assay provides a scalable method for measuring residual HIV viremia that may be broadly useful in pathogenesis and cure-directed studies.

**IMPORTANCE:**

Most people with HIV (PWH) on antiretroviral therapy have viral loads below the detection limit of clinical assays, yet virus is often present and detectable at very low levels using ultrasensitive research assays. Clinical trials evaluating curative interventions and interpreting outcomes of analytical treatment interruptions depend on reliable assays to assess and quantify changes in HIV persistence often at very low levels. We conducted a two-stage head-to-head, blinded comparison of multiple ultrasensitive HIV RNA and p24 assays, first using 50 low viral load plasma samples, then further evaluating the top-performing assays on a 144-member blinded panel composed of duplicate contrived and clinical specimens from well-suppressed PWH. Single-copy RNA methods performed better than p24 assays, and a fully automated, 9-replicate commercial RNA assay demonstrated high sensitivity, reproducibility across laboratories, and practical scalability that can be applied to measure the impact of interventions in HIV cure trials.

## INTRODUCTION

Antiretroviral therapy (ART) suppresses HIV viremia to levels that are undetectable by clinical viral load assays and (generally) requires lifelong adherence to ART to sustain viral suppression. However, single-copy assays can detect HIV RNA well below the limit of detection of clinical assays (1, 2). Low levels of HIV in plasma reflect viral persistence, largely due to cells that were infected near the time of ART introduction, subsequently resulting in clonal or oligoclonal proviruses that, following discontinuation of ART, become highly transcriptionally active and continue to release viral particles (3–5). In some people, viremia can persist at a level detectable by clinical viral load assays, despite optimal ART adherence and absence of HIV drug resistance. This non-suppressible viremia (NSV) can arise from proviruses with small deletions and mutations that affect the major splice donor site in the 5′ leader region (6, 7).

In the quest for a cure for HIV, many interventional clinical trials are underway utilizing various strategies that target the latent reservoir in ART-suppressed participants (8). The HIV reservoir can be quantified by assessing the levels and replication competence of proviruses in peripheral blood mononuclear cells (PBMC), such as total HIV DNA and intact proviral DNA, as well as by expression of viral RNA, protein, and replication competence based on viral outgrowth assays (9). One strategy used to assess the effect of a curative intervention on the replication-competent reservoir is the use of analytical treatment interruption (ATI), with measurement of time to and/or magnitude of plasma viral rebound following ATI. Timing of HIV viral rebound has been correlated with timing of ART initiation, with the strongest predictor of faster time to rebound being higher residual plasma viremia before treatment interruption in early-treated individuals and higher levels of intact proviral DNA in individuals treated during chronic infection (10). Theoretically, circulating virus—which is measured by plasma RNA tests—reflects the total body burden of virus-producing cells. Thus, plasma RNA tests that sample and quantify circulating virus reflective of the clinically relevant HIV reservoir are increasingly appreciated as a key measure of the impact of curative interventions.

The Reservoir Assay Validation and Evaluation Network (RAVEN) program was developed to establish performance characteristics and limitations of currently available and novel assays for detection and quantitation of the HIV reservoir in blood of ART-suppressed individuals, with the goal of informing their application to selection and monitoring of ART-suppressed HIV-infected populations of interest for cure interventions, including those incorporating ATI. Given that different anatomical and cellular compartments can harbor persistent virus, and that currently available and novel assays measure various aspects of the HIV provirus and its functionality, the RAVEN program included evaluations of the quantitative viral outgrowth assay (QVOA) and other viral outgrowth assays for measuring the replication-competent HIV reservoir in PBMC (11–14), assays for measuring levels of intact or inducible viral nucleic acid or protein in PBMC (15), and ultrasensitive assays for residual viremia in plasma (16). The latter study focused on the performance of a fully automated replicate testing assay for HIV RNA, demonstrating that persistent low-level viremia can be quantified in individuals on ART and that viral levels decline with length of time on suppressive ART.

Here, addressing the RAVEN program objective to evaluate ultrasensitive assays for residual viremia in plasma, we describe a head-to-head comparison of multiple ultrasensitive assays for detection of HIV RNA or p24 antigen in plasma using blinded panels of serially diluted analytic standards, clinical samples from PWH on ART with viremia undetectable by standard clinical viral load assays, and samples from people without HIV. The study consisted of two phases, beginning with a qualification phase that evaluated five HIV RNA and two p24 assays with a smaller panel, followed by an evaluation phase on the most sensitive assays with a larger panel.

## MATERIALS AND METHODS

### Sample selection and panel construction

RAVEN qualification phase plasma panels were built separately for HIV subtypes B and C, each including 50 samples consisting of (i) low viral load plasma samples from 20 PWH characterized as HIV RNA-negative antibody (Ab)-positive, (ii) five-step threefold serial dilutions in duplicate of two well-characterized HIV RNA-positive/Ab-negative plasma samples diluted in HIV-negative human serum, and (iii) plasma samples from 10 people without HIV (Table 1). The HIV-positive samples were obtained from plasma units acquired through regular blood donations and identified by pooled (for subtype B) or individual (for subtype C) nucleic acid testing, as well as by serology testing as HIV RNA-positive/Ab-negative or RNA-negative/Ab-positive. Although plasma samples were not tested to determine HIV subtype, it is highly likely that they consisted of subtype B and subtype C infections based on prevalence within the geographic location of blood collection sites in the USA and South Africa, respectively (17). Blood donations were collected with ethical review and approval of the collection protocol and informed consent as previously established for the donor organizations’ collection activities. For HIV-positive samples identified as RNA-negative/Ab-positive through blood donor screening, additional testing was performed using (i) 10 to 20 replicates of a nucleic acid testing assay used in the blood bank setting to determine specimen reactivity for HIV and (ii) nine replicates of the Aptima HIV-1 Quant Dx assay. A total of 20 samples (each for subtypes B and C) were selected, representing a range of low viral loads, based on reactivity in at least one replicate during the additional testing. RNA-positive/Ab-negative samples were quantified using the Abbott *m*2000 RealTi*m*e HIV-1 Viral Load assay at an intermediate dilution 1 log_10_ above the highest concentration to be included in the panel, prior to preparing the final serial dilutions to nominal concentrations of 27 to 0.3 copies/mL in defibrinated human plasma (Gemini Biosciences). All samples were frozen into 5 mL aliquots at −80°C and assembled into blinded panels.

**TABLE 1:** Composition of samples in qualification and evaluation phase plasma panels^*c*^

Sample type	# of participant samples	Replicates	Nominal cp/mL
Qualification phase^*a*^ *N* = 50			
HIV RNA-/Ab+	20	Singlicate	
Analytic standards			
HIV RNA+/Ab-	2	Duplicate	0.3, 1, 3, 9, 27
HIV-negative	10	Singlicate	
Evaluation phase^*b*^ *N* = 144			
HIV+ ART-suppressed	20 at two time points	Duplicate	
HIV+ non-suppressed			
Elite controllers	2	Duplicate	
Non-suppressed	3	Duplicate	
Analytic standards			
HIV RNA+/Ab-	1	Duplicate	Standard 1: 0.3, 1, 3, 9, 27
	1	Duplicate	Standard 2: 0.45, 1.4, 4.5, 14, 45
HIV+ non-suppressed	One spiked in two diluents		
	HIV-negative diluent	Duplicate	Standard 3: 0.45, 1.4, 4.5, 14, 45
	HIV+ aviremic diluent	Duplicate	Standard 4: 0.45, 1.4, 4.5, 14, 45
Diluent	HIV-negative diluent	Duplicate	
	HIV+ aviremic diluent	Duplicate	
HIV-negative	5	Duplicate	

^*a*^ Two separate panels were constructed: one HIV subtype B and one HIV subtype C.

^*b*^ HIV subtype B only.

^*c*^ Ab, antibody.

For the RAVEN evaluation phase, samples were collected via apheresis (with specific consent for collections and testing, as approved by the UCSF Committee on Human Research), processed, and stored from a well-characterized UCSF cohort of PWH with HIV-1 subtype B infection who had started ART ≤6 months (early) or ≥1 year (late) after infection and had been well-suppressed for ≥1 year, with an average of two apheresis collections approximately one year apart (16). The cohort also included elite controllers (PWH in whom viral suppression is maintained for several years in the absence of ART), non-suppressed PWH, and people without HIV. These samples were used to construct 144-member blinded subtype B plasma evaluation panels, consisting of duplicate aliquots of (i) low viral load clinical samples from early-treated (*n* = 10) and late-treated (*n* = 10) PWH (each at two visits), (ii) clinical samples from untreated elite controllers (*n* = 2) and non-suppressed participants (*n* = 3), (iii) analytic standards of five-step half-log_10_ serially diluted HIV RNA-positive/Ab-negative plasma and of HIV viremic plasma, and (iv) HIV-negative plasma (Table 1). Two sets of analytic standards were included. Standards 1 and 2 were derived from serially diluted HIV RNA-positive/Ab-negative plasma. Standard 1 was identical to that used in the subtype B qualification phase panel (nominal concentrations of 27 to 0.3 copies/mL), and Standard 2 had concentrations of 45 to 0.45 copies/mL. Standards 3 and 4 were serially diluted HIV+ non-suppressed plasma to concentrations of 45 to 0.45 copies/mL in 2 diluents; Standard 3 in commercial HIV-negative defibrinated human plasma and Standard 4 in pooled aviremic plasma from recalled fully-suppressed participants with undetectable HIV RNA (based on 45-replicate Hologic Aptima HIV-1 Quant Dx assay). All samples were frozen into 5 mL aliquots at −80°C and assembled into blinded panels.

Single-copy assay controls obtained from the Virology Quality Assurance (VQA) program (contract # HHS75N93019C00015) at Duke University were quantified by the Roche cobas HIV-1 test, then spiked and diluted into HIV-1 negative plasma at 20, 5, and 0 copies/mL. For each concentration, 18 aliquots (1.8 mL each) were randomly selected across the entire production of each control level for testing using the three-replicate Hologic Aptima HIV-1 Quant Dx assay.

### Single-copy HIV RNA assays

The gSCA, HMMC gag, iSCA, and iSCA v2 assays were performed as previously described (2, 18–20). The gSCA assay is performed using a primer set targeting the 5′ end of *gag*, whereas the iSCA (v1 and v2) assay targets the 3′ region of *pol*. The iSCA primer and probe binding sites were selected in a highly conserved sequence in the integrase region; sequence conservation agreement was excellent across subtypes, except for subtype C, where a one-nucleotide substitution was present in the iSCA forward primer-binding site. Therefore, the subtype C panel was not tested using the iSCA assay. For the HMMC gag assay, samples were centrifuged for 30 min, 10°C at 43,100 × *g* (Beckman OPTIMA XE-90IVD with Ty70.1Ti rotor), and nucleic acids were isolated and analyzed as described previously (2, 21) using the *gag* primer set designed for the gSCA assay, as well as additional primers and probe described in Somsouk et al. (18), in which the forward primer spans the major splice donor site in the 5′ leader region. For the ultrasensitive Roche CAP/CTM assay, samples provided were centrifuged for 1 h at 41,860 × *g* (Sorvall RC6 + with rotor model SM-24) at 4°C. From the 4.9 mL total volume, 3.7 mL of plasma supernatant was removed from each sample, leaving 1.2 mL behind (Table 2). Then, the remaining pellet volume was tested using 1.1 mL of sample on the COBAS AmpliPrep/COBAS TaqMan HIV-1 Test, v2.0, per the package insert, targeting two regions, *gag* and the long terminal repeat (LTR). The concentration of each sample was corrected by a factor of 4.08 (4.9 mL/1.2 mL), wherein the raw result was divided by 4.08 to correct for the total volume originally used in the assay. For the Automated 18×, 9×, and 3× assays, replicates (18, 9, or 3 replicates, respectively) of the Aptima HIV-1 Quant Dx assay on the Panther platform were performed according to the manufacturer instructions, targeting two regions: *pol* and 5′ LTR. The Panther instrument was programmed to generate up to nine independent replicate tests for each specimen aliquot tube containing up to 5 mL, with each replicate test processing 0.5 mL input. Quantification was based on Poisson modeling of the number of negative (at least one) out of total replicates tested. For samples where no negative replicates were observed, viral load for each replicate was imputed using the assay’s normal calibration combined with offline analysis, and then averaged across all replicates (16, 22). For testing the VQA single-copy assay controls using the Automated 3× assay, the algorithm for estimating viral load from replicate measurements is provided in the Supplemental Materials.

**TABLE 2:** Assays used to measure low copy HIV in qualification and evaluation phase plasma panels

Assay	Testing labs	Sample processing
Qualification phase (subtype B and C panels)		
Nucleic acid assays		
Single-copy real-time RT-PCR targeting gag (gSCA)	University of Sydney	Ultracentrifugation
Triplex qRT-PCR based on gSCA with additional gag-based assay (HMMC gag)	Frederick National Lab for Cancer Research	Ultracentrifugation
Single-copy real-time RT-PCR targeting integrase (iSCA)^*a*^	University of Pittsburgh	Ultracentrifugation
Pre-analytically modified Roche CAP/CTM HIV-1 test, v2.0	Rush University Medical Center	Ultracentrifugation
18× replicate testing with Hologic Aptima HIV-1 Quant Dx assay	University of California San Diego	None
Antigen assays		
Quanterix Simoa ultrasensitive p24 immunoassay	Merck & Co., Inc., Rahway, NJ, USA	None
MSD S-PLEX ultrasensitive p24 immunoassay	MSD	Acid/base treatment
Evaluation phase (subtype B panel)		
HMMC gag	Frederick National Lab for Cancer Research	Ultracentrifugation
iSCA v2	University of Pittsburgh	Micro-centrifugation
9× replicate testing with Hologic Aptima HIV-1 Quant Dx assay	Vitalant Research Institute, University of Pittsburgh, Accelevir	None

^*a*^ Only the subtype B panel was tested using the iSCA assay during the qualification phase.

### Ultrasensitive p24 antigen assays

Testing using the Quanterix Simoa HIV p24 assay was performed as previously described (23). The Meso Scale Discovery (MSD) S-PLEX^®^ HIV p24 assay was performed as described by Stengelin et al. (24). In the current study, samples underwent acid dissociation of immune complexes and 40-fold concentration prior to detection. Four milliliters of each blinded plasma sample from the RAVEN qualification phase panel, as well as positive and negative plasma control samples (prepared by MSD), were treated with 2 mL of HCl, followed by neutralization with 2 mL of HEPES-buffered NaOH. The volume was then reduced to 100 µL using capture agents that have reversible binding (Table 2). Concentrated samples were frozen at −80°C and later tested in batches in a randomized and blinded fashion. An eight-point calibration curve, run in duplicate, was included on each plate, and the data were fitted with a weighted four-parameter logistic curve fit. Concentrations of each sample were calculated from the calibrator curves, taking into account sample pretreatment. The mean of two measurements was derived for each sample.

### Statistical analysis

For the RAVEN evaluation phase, probit modeling was used to estimate the concentration, where the probability of detection of HIV in analytic standards is 50% (LOD_50_) for each assay. The probit models were fitted using the R drc package. The estimated means and 95% confidence intervals of the LOD were calculated.

Assay sensitivity and precision were compared relative to the reference HMMC gag assay. This assay was selected as the benchmark due to its well-established application in many clinical trials. Sensitivity was defined as the probability that an assay returns a positive result when there is a single HIV RNA copy present in the sample assayed. Specifically, we estimated the ratios of the probability of detecting a positive result for an assay to that of a reference assay (HMMC gag) by fitting a Poisson regression model. The detailed method for assessing relative sensitivity and precision is provided in the Supplemental Materials.

Separately, to assess reproducibility of the Automated 9× assay across the three separate testing laboratories, we first calculated pairwise (Labs 1 and 2; Labs 1 and 3) interlab variances and means, and then derived pairwise interlab CVs using equation (3) from the same section. Finally, we estimated the relative pairwise interlab precision by taking the logarithm of the ratio of the two pairwise interlab CVs, with P-values for the null hypothesis of equal interlab variation.

## RESULTS

### Performance of ultrasensitive HIV RNA assays using the RAVEN qualification phase plasma panels

**Fig 1:**
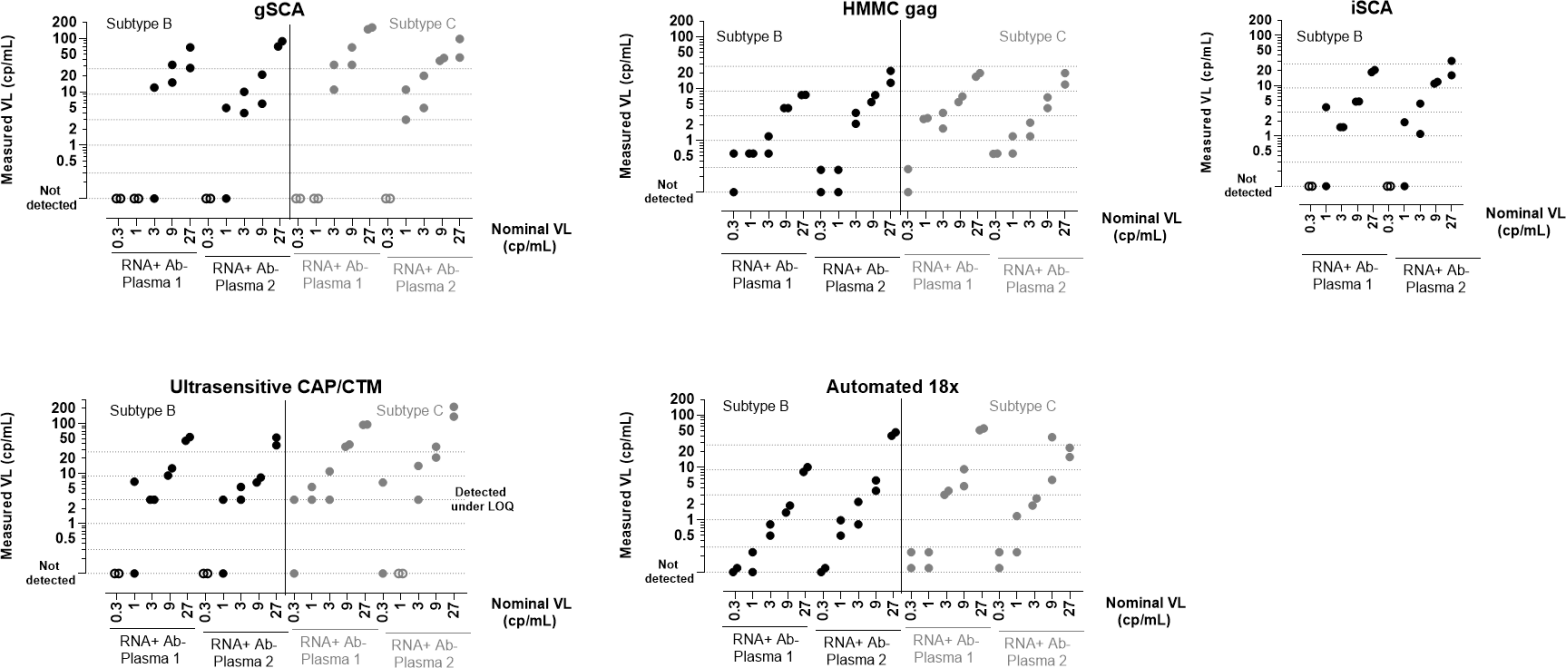
Nominal and measured viral load (VL) using single-copy assays in qualification phase plasma panels. For each HIV subtype (B and C), plasma from two different individuals, identified as HIV RNA-positive and Ab-negative through routine blood donor testing, was serially diluted and blinded, and each concentration was included in duplicate in the panel for measurement of VL using single-copy assays. Open symbols indicate that HIV was not detected in both replicates using the single-copy assay. LOQ, limit of quantification.

The HMMC gag and Automated 18× assays detected the lowest nominal concentration (0.3 cp/mL) in at least one of the duplicate aliquots for both subtype-specific analytic standards, whereas the ultrasensitive CAP/CTM assay detected the lowest concentration in one of the duplicates for both subtype C analytic standards, but not in either of the subtype B analytic standards. The gSCA and iSCA assays were unable to detect the 0.3 cp/mL concentration (Fig. 1). At 1 cp/mL, the HMMC gag, iSCA, and Automated 18× assays detected at least one of the duplicates for all analytic standards tested. The ultrasensitive CAP/CTM assay did not detect the 1 cp/mL concentration in one of the two subtype C analytic standards, while the gSCA assay did not detect this concentration in one each of the two subtype-specific analytic standards. At nominal concentrations ≥3 cp/mL, all five single-copy assays detected HIV in the analytic standards. The reported viral loads were higher at higher nominal HIV RNA concentrations.

Specificity was evaluated using plasma samples from 10 people without HIV per panel. For the HMMC gag, iSCA, ultrasensitive CAP/CTM, and Automated 18× assays, 100% specificity was observed. For the gSCA assay, false positives were observed for 3 of 10 negative controls included in the subtype C panel (at measured HIV RNA concentrations of 1, 1, and 13 cp/mL), whereas no false positives were observed for the same 10 negative controls included in the subtype B panel.

**Fig 2:**
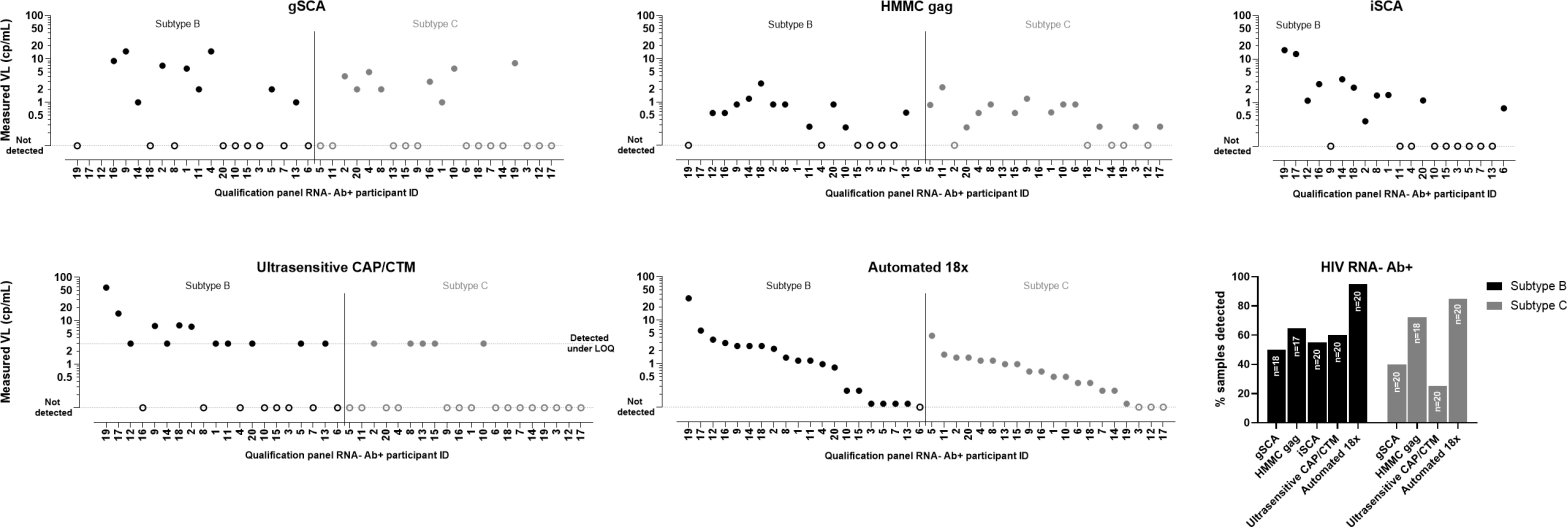
Measured VL in plasma samples from individuals identified as HIV RNA-negative and Ab-positive through routine blood donor testing. For each HIV subtype (B and C), plasma samples were blinded and included in singlicate in qualification phase panels for testing using single-copy assays. Open symbols indicate that HIV was not detected. Symbols were left blank if the measurement was invalid for a sample. Participant ID samples are ordered from highest to lowest measured VL using the Automated 18× assay. In the bar graph of proportion of samples detectable by single-copy assay, the number of samples with valid measurements is shown inside the bars.

Detection of HIV in clinical samples was compared using 20 subtype-specific plasma samples from HIV-positive individuals with undetectable HIV RNA by blood donation testing. The Automated 18× assay had the highest clinical sample detection frequency, with 95% and 85% detection of subtype B and subtype C clinical samples, respectively (Fig. 2). Detection frequencies for the subtype B clinical samples were 65%, 60%, 55%, and 50% for the HMMC gag, ultrasensitive CAP/CTM, iSCA, and gSCA assays, respectively, whereas for the subtype C clinical samples, they were 72%, 40%, and 25% for the HMMC gag, gSCA, and ultrasensitive CAP/CTM assays, respectively.

### Performance of ultrasensitive HIV p24 assays using the RAVEN qualification phase plasma panels

The Simoa ultrasensitive p24 assay detected HIV antigen above the cutpoint at all concentrations of the analytic standards; however, the detected p24 antigen levels and the nominal HIV RNA concentrations were not correlated (Fig. S1). The S-PLEX ultrasensitive p24 assay did not detect any of the analytic standards, except for one of the two 1 cp/mL replicates for one of the subtype C analytic standards.

No detection of HIV in plasma samples from HIV-uninfected individuals was observed using the S-PLEX p24 assay, whereas HIV was detected (at 5.6 fg/mL) in 1 of 10 negative controls using the Simoa p24 assay. Low detection frequencies were observed for the clinical samples, with 10% of subtype B and 20% of subtype C samples detected above the cutpoint for the Simoa p24 assay, while the S-PLEX p24 assay detected 5% of subtype B and 10% of subtype C clinical samples.

### Ultrasensitive HIV RNA assay evaluation

Based on assay performance, with the highest detection frequencies in the RAVEN qualification phase plasma panels, the HMMC gag, iSCA, and Automated 18× assays were chosen for further testing in the RAVEN evaluation phase plasma panels. In this phase, the iSCA v2 assay, with higher sensitivity and simpler sample processing (20), was performed in lieu of the iSCA v1 assay. In addition, the Automated 18× assay was replaced with the Automated 9× assay to match the sample volume (5 mL rather than 10 mL) used for the other two single-copy assays, allowing for a more direct comparison. Whereas the HMMC gag and iSCA v2 assays rely on manual methods involving multiple steps, the Automated 9× assay is based on replicate testing using a commercial automated platform. Therefore, this assay was performed in three different laboratories to assess reproducibility of the results.

**Fig 3:**
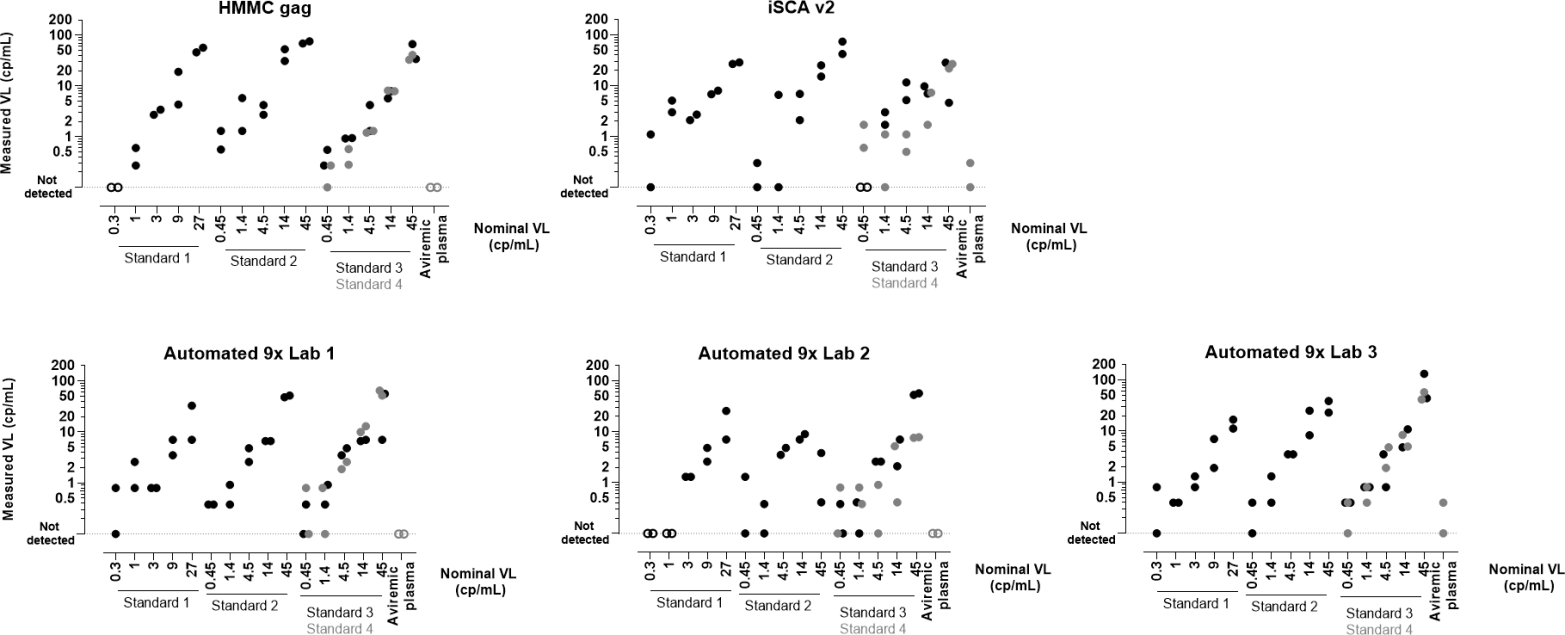
Nominal and measured VL using single-copy assays in evaluation phase plasma panels. Plasma from two different individuals, identified as subtype B HIV RNA-positive and Ab-negative through routine blood donor testing, was serially diluted, blinded, and included in the panel in duplicate for each concentration. Plasma from an HIV-positive RAVEN participant with high VL was serially diluted in two different diluents (with or without anti-HIV antibodies), and each concentration was included in duplicate in the panel. Samples diluted in HIV-positive aviremic plasma are indicated in gray compared to samples diluted in HIV-negative defibrinated plasma. Open symbols indicate that HIV was not detected in both replicates using the single-copy assay.

At the lowest nominal concentration (0.3 cp/mL) for analytic Standard 1 (that was included in both the subtype B qualification phase panel and the evaluation phase panel), detection was observed for one of the duplicate aliquots using the iSCA v2 assay and the Automated 9× assay in two of the three testing labs (Fig. 3), whereas no detection was observed using the HMMC gag assay and the Automated 9× assay in one of the testing labs (where no detection was observed for the 1 cp/mL nominal concentration as well). All assays detected HIV in at least one of the two duplicate aliquots in analytic Standards 2 and 3 at the lowest nominal concentration (0.45 cp/mL), except for the iSCA v2 assay in Standard 3.

To determine whether fibrin or other insoluble complexes in plasma impair nucleic acid isolation for HIV recovery by the different single-copy assays, we assessed HIV detection sensitivity in standards diluted in commercial defibrinated plasma and in aviremic plasma, with no difference in detectability observed for any of the assays (Fig. 3). In the aviremic HIV+ plasma diluent, detection was not observed (by HMMC gag and Automated 9× in two of the three testing labs) or was observed for one of the duplicate aliquots at the assay limit of detection of 0.30 to 0.38 cp/mL for iSCA v2 and Automated 9×.

The LOD_50_ for detection sensitivity on the analytic standards was calculated for each assay, resulting in similar estimates (Table 3). For the two manual assays, HMMC gag and iSCA v2, the LOD_50_ was 0.4 cp/mL (95% CI, 0.34 to 0.45 cp/mL) and 0.41 cp/mL (95% CI, 0.02 to 0.8 cp/mL), respectively. For the Automated 9× assay, the LOD_50_ was 0.25 cp/mL (95% CI, 0 to 0.51 cp/mL), 0.94 cp/mL (95% CI, 0.17 to 1.7 cp/mL), and 0.27 cp/mL (95% CI, 0.01 to 0.52 cp/mL) in Lab 1, Lab 2, and Lab 3, respectively.

**TABLE 3:** LOD_50_ of assays used to measure low copy HIV in evaluation phase plasma panels

Assay	LOD_50_ in cp/mL (95% CI)
HMMC gag	0.4 (0.34, 0.45)
iSCA v2	0.41 (0.02, 0.8)
Automated 9× (Lab 1)	0.25 (0, 0.51)
Automated 9× (Lab 2)	0.94 (0.17, 1.7)
Automated 9× (Lab 3)	0.27 (0.01, 0.52)

Specificity was evaluated in five HIV-negative clinical samples and commercial HIV-negative defibrinated human plasma, each in duplicate, with 100% specificity observed for the iSCA v2 and Automated 9× assays in two of the three testing labs. False positives were observed for one of two replicates of one HIV-negative clinical sample on the HMMC gag assay (at the assay limit of detection of 0.26 cp/mL) and the Automated 9× assay in one of the testing labs (at the assay limit of detection of 0.38 cp/mL).

**Fig 4:**
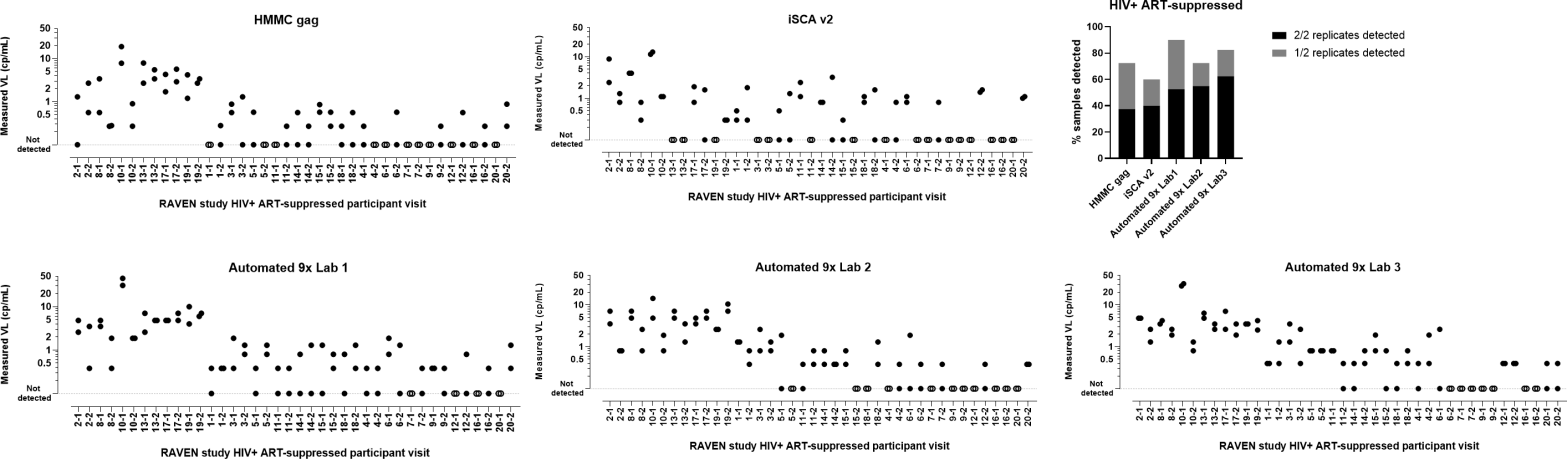
Measured VL in plasma samples from HIV-positive RAVEN participants on antiviral therapy. For each participant, plasma samples from two different visits, approximately one year apart, were each blinded and included in duplicate in the evaluation Ppase panel. Open symbols indicate that HIV was not detected in both replicates using the single-copy assay. The stacked bar graph of proportion of samples detectable by single-copy assay indicates whether HIV was detected in both replicates or in only one of the duplicates for each participant visit sample.

Detection of clinical samples was evaluated on 40 plasma samples from 20 HIV+ ART-suppressed RAVEN participants, each at two visits approximately one year apart. The Automated 9× assay had the highest clinical sample detection frequency, with 90% (52.5% in both replicates), 82.5% (62.5% in both replicates), and 72.5% (55% in both replicates) of samples detected in at least one of the two replicates for the three different testing labs (Fig. 4). The HMMC gag assay detected 72.5% of the samples (37.5% in both replicates), whereas the iSCA v2 assay detected 60% of the samples (40% in both replicates).

Plasma from two elite controller RAVEN participants was detectable on all assays, except for one Automated 9× assay testing lab which failed to detect one sample (Fig. S2). All assays demonstrated good reproducibility of viral load on both replicates of three non-suppressed RAVEN participant samples and were within approximately fivefold across the assays, except for the iSCA v2 assay which quantified viral load two orders of magnitude lower than that for the other assays on one non-suppressed participant sample.

Assay sensitivity was compared using statistical modeling of the results from the diluted analytical standards of the clinical samples as well as the low viral load clinical samples from treated PWH and the clinical samples from untreated elite controllers. The HMMC gag assay, which has been applied to a large number of clinical studies, was used as the benchmark for comparison. Sensitivity of the iSCA v2 assay did not differ significantly relative to that of the HMMC gag assay, whereas the sensitivity of the Automated 9× assay was higher relative to that of the HMMC gag assay. For Lab 1 and Lab 3, sensitivity was 1.6-fold (95% CI, 1.1-fold to 2.3-fold) and 1.8-fold (95% CI, 1.2-fold to 2.5-fold) higher, respectively. For Lab 2, the difference was not statistically significant (Fig. S3).

Precision was compared by estimating the ratios of the assays’ CVs using HMMC gag as the reference assay. Thus, the CV of the iSCA v2 assay did not differ significantly from that of the HMMC gag assay, whereas the CV of the Automated 9× assay was lower relative to that of the HMMC gag assay. For Labs 1, 2, and 3, CV was 1.3-fold (95% CI, 1.1-fold to 1.6-fold), 1.3-fold (95% CI, 1.1-fold to 1.5-fold), and 1.3-fold (95% CI, 1.1-fold to 1.5-fold) lower, respectively.

Reproducibility of the Automated 9× assay across the three labs was assessed by estimating pairwise interlab CVs, with no significant difference found in reproducibility across the three labs.

### Characterization of Virology Quality Assurance single-copy assay standards

The Virology Quality Assurance (VQA) program prepares and distributes standards consisting of laboratory-cultured HIV virions diluted in plasma to low concentrations, for use in assessing assays that measure low-level HIV viremia in PWH on ART in HIV cure trials. To verify low-copy-number concentrations (0, 5, and 20 cp/mL) and homogeneity across aliquots of a VQA production batch of single-copy assay controls, 18 aliquots of each concentration were tested. Each 1.8 mL aliquot was tested in triplicate (Automated 3× assay).

**Fig 5:**
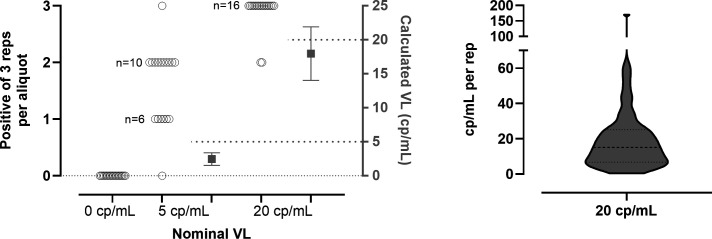
Measured VL in VQA SCA controls (negative, 5 copy/mL, 20 copy/mL) using automated 3× assay in 18 randomly chosen aliquots for each control. The calculated VL for the 5 cp/mL control was derived using the low-copy-number algorithm, based on 29 positives of 54 reps. For the 20 cp/mL control, the calculated VL was measured by removing the outlier (at 170 cp/mL) and deriving the arithmetic mean and 95% CI for the remaining 51 positive reps. Error bars around the calculated VL depict the 95% CI. The violin plot in the right panel depicts the VL for the 52 individual positive replicates across all 18 aliquots of the 20 cp/mL control.

All 54 replicates of the 0 cp/mL control were not detected. Testing of the 5 cp/mL control resulted in two of three reps detected for 10 aliquots, and one of three reps detected for six aliquots, with the remaining two aliquots having no detection or all three reps detected (Fig. 5). The majority (16 aliquots) of the 20 cp/mL control showed detection in all three reps, while two aliquots showed detection in two of three reps. The distribution of positive replicates across aliquots at each concentration suggests acceptable homogeneity. The concentration of the 5 cp/mL control was inferred at 2.46 cp/mL (95% CI 1.55, to 3.38 cp/mL) based on detection in a total of 29 of 54 reps (Supplemental Materials). The 20 cp/mL control concentration was calculated at 17.98 cp/mL (95% CI, 14.04 to 21.91 cp/mL).

## DISCUSSION

Here, we evaluated the performance characteristics of ultrasensitive assays for HIV RNA and p24 antigen in plasma using blinded panels containing contrived specimens prepared from acutely infected blood donor plasma, clinical samples with low levels of HIV RNA from unsuppressed and virally suppressed PWH on ART, and samples from HIV uninfected individuals. These ultrasensitive research assays are essential tools in HIV curative interventions in monitoring for viral rebound after treatment interruption, providing a reliable baseline against which post-intervention outcomes can be compared and identifying subtle differences in rebound kinetics among individuals (25–27), as well as for confirming HIV infection and quantifying low-level viremia during follow-up in cases of breakthrough HIV infection in people on PrEP (28, 29).

Most of the assays employed in this study had previously been described in publications detailing their development and validation. The original single-copy assay, gSCA (2), detected concentrations down to 0.78 cp/mL using HIV virions diluted in human plasma and measured concentrations ranging from 1 to 32 cp/mL in plasma samples (7 mL volume) from 15 of 15 HIV-infected ART-suppressed individuals. However, a comparison of the gSCA assay to a commercial HIV viral load assay for plasma samples from 20 untreated individuals with viral load >1,000 cp/mL showed that 15% of samples were markedly under-quantified by the gSCA assay, suggesting that genetic variation in HIV *gag* affected amplification from clinical samples using the single-copy assay. Nonetheless, the gSCA assay has been successfully applied to measure residual viremia in several seminal studies of HIV persistence and clinical interventions (30–33).

The HMMC gag assay was designed based on the gSCA assay with the addition of primer sets targeting an additional *gag* region (18). The amplification reactions are set up in 12 replicates, with quantification based on standard curve interpolation (where all replicates were positive) or application of Poisson distribution analysis (where fewer than 12 replicates were positive). Using this assay on plasma samples (9 mL volume) from 33 ART-suppressed participants in a clinical trial, 91% of participants had detectable viremia (median 0.6 and 2.1 cp/mL at baseline in the two arms of the trial [18]). The HMMC gag assay has been applied to numerous clinical trials and studies of HIV persistence and cure interventions on the HIV reservoir (18, 27, 34–48).

Another assay, iSCA (19), targeting the conserved integrase region in the *pol* gene, rather than *gag*, was developed to decrease the potential for mismatches of primer sets with sequences from clinical samples. In addition, nucleic acid recovery was improved by modifying the sample processing after ultracentrifugation through addition of a chaotropic agent during protein digestion and other changes to subsequent steps. Comparison of iSCA and gSCA assays on plasma samples (3 mL volume) from 25 ART-suppressed individuals showed that 36% and 16% had concordant detectable and undetectable viremia, respectively. The remaining were detectable only by iSCA (32%) or gSCA (16%). Similar to other single-copy assays, the iSCA assay has been applied to numerous studies assessing the impact of HIV curative interventions or association of host characteristics with residual viremia (49–51).

Subsequently, iSCA v2 (20) was developed as a simpler and more sensitive assay based on iSCA v1 using microcentrifugation rather than ultracentrifugation and assaying a larger fraction of the total nucleic acid extract. A rigorous assessment of the iSCA v2 95% limit of detection estimated it to be 0.994 cp/mL (95% CI, 0.62 to 2.55 cp/mL) for a 5 mL volume of low-copy-number standards of diluted plasma from a viremic HIV-positive individual. Furthermore, testing of plasma samples (1 to 5 mL volume) from 60 ART-suppressed individuals demonstrated that 45% had detectable viremia by both iSCA v1 and v2, 23% had undetectable viremia by both assays, 28% had viremia detectable only by iSCA v2, and 3% had viremia initially detectable only by iSCA v1, although these samples were detectable by iSCA v2 upon subsequent testing. The iSCA v2 assay has been employed in investigations of the HIV reservoir in a clinical case of ART-free remission following transplantation with CCR5Δ32/Δ32 stem cells (52), as well as rare cases of HIV infection identified in the treatment arm of PrEP clinical trials (53).

The gSCA, HMMC gag, and iSCA (both v1 and v2) assays all rely on manual centrifugation and nucleic acid extraction steps with low throughput. Consequently, modified assays using automated HIV viral load assay platforms have been utilized to allow higher throughput. The modifications include sample concentration through centrifugation on a density cushion (54) or ultracentrifugation (55) combined with extraction and amplification using commercial tests, such as the Abbott RealTi*m*e HIV-1 assay, Roche CAP/CTM HIV-1 test (as described in this study), and Hologic Aptima HIV-1 Quant Dx assay (22). Alternatively, a multi-replicate strategy, rather than sample concentration, has been reported for the Aptima HIV assay (16, 22, 56, 57). Bakkour et al. developed a Poisson modeling method to calculate HIV RNA concentration based on the combination of positive and negative replicates. Using the Automated 45× replicate assay, HIV RNA was detected (median 0.54 cp/mL) in plasma from each of 50 individuals within the RAVEN cohort (16). Furthermore, in that study, no detection was observed in 447 replicates with valid results from plasma samples from 10 people without HIV. Jacobs et al. performed a comparison of the iSCA v2, Automated 1× concentrated, and Automated 9× replicate assays, demonstrating that the iSCA v2 method had the highest variation and lowest 95% LOD. The Automated 1× concentrated method had reduced analytic sensitivity and was not further evaluated for clinical sensitivity. The latter was evaluated using plasma samples from 50 ART-suppressed individuals, showing higher detection frequency with Automated 9× Aptima assay (82%) than iSCA v2 (62%).

Thus, based on prior studies comparing single-copy HIV RNA assays, clinical sensitivity was higher for iSCA compared to gSCA, for iSCA v2 compared to iSCA v1, and for Automated 9× compared to iSCA v2. To our knowledge, this study is the first head-to-head comparison of multiple ultrasensitive assays for detection of residual HIV in plasma. In addition to assays detecting HIV RNA, we included ultrasensitive HIV p24 assays due to their small volume requirements, high throughput, and lower cost (58–61). The ultrasensitive p24 assays have been used to measure HIV *gag* p24 protein in plasma from acutely infected HIV-positive individuals. However, their application to samples from ART-suppressed individuals has focused on CD4^+^ T cells (14, 23, 62–64) rather than plasma, due to the formation of immune complexes after seroconversion during chronic infection.

Based on the results of this study, the ultrasensitive p24 assays, as performed, were not suitable for detection in plasma samples from HIV Ab-positive individuals with residual low-level viremia and may require enhancements to the methodology to improve detection and reduce background. Such improvements have been developed to enrich p24 through immunocapture and elute in assay-compatible format, allowing sensitive and specific detection in lysates of cells from ART-suppressed HIV-positive individuals (65–67).

We showed herein that the gSCA assay had lower clinical sensitivity on subtype B plasma samples than the iSCA, HMMC gag, and ultrasensitive assays using commercial HIV tests, confirming and extending previously reported findings. We also found that the clinical sensitivity of the iSCA v2 assay was lower than that of the HMMC gag and Automated 9× assays. For subtype C plasma samples, the gSCA assay had higher clinical sensitivity than the ultrasensitive CAP/CTM assay, but lower than the HMMC gag and Automated 18× assays. It should be noted that the CAP/CTM system has been replaced with higher throughput systems (such as the cobas 6800/8800 platform) in many laboratories. The newer system has been reported to have higher sensitivity in detecting residual viremia in ART-suppressed individuals for both B and non-B subtypes (68). In the current study, the use of the high-throughput Automated 9× assay in three different laboratories showed reproducible results. The wide availability of such automated systems has led to their use in current global clinical trials networks, such as the AIDS Clinical Trials Group (10), and in other multi-center studies, such as those performed by the Martin Delaney Collaboratories for HIV Cure Research (69).

In this study, clinical sensitivity was evaluated using plasma samples from PWH with undetectable viremia using clinical assays. As noted earlier, non-suppressible viremia has been shown to arise from proviruses containing defects in the 5′ leader sequence. It is possible that such defective RNA contributes to residual viremia in people receiving suppressive ART. The single-copy HIV RNA assays employed in the current study target regions outside of the 5′ leader defective sequences and/or have dual target regions. Therefore, it is unlikely that such mutations in the 5′ leader sequence would affect the performance of the single-copy assays on the clinical samples included in the panels.

Further evaluation of assay performance on HIV subtype C and other non-B subtypes was not performed in this study and warrants further investigation, given the geographic distribution of these subtypes in regions where clinical trials are being undertaken worldwide. Of note, the iSCA v2 assay requires an alternative primer set for adequate quantification of subtype C isolates (22). We found that both the HMMC gag and the Automated 18× assays had good clinical sensitivity (72% and 85%, respectively) using subtype C samples from blood donors with undetectable HIV RNA using routine screening assays. Another limitation of our study consists of a lack of further comparison between commercial assays with pre-processing by centrifugation and manual or replicate single-copy assays. Although ultracentrifugation followed by Automated 1× assay had been investigated in a prior study (22) and found to have lower analytic sensitivity than the other single-copy assays tested, we found that the ultrasensitive CAP/CTM assay had reasonable clinical sensitivity (60%) on subtype B samples. Generating additional comparative data on the widely used commercial viral load platforms would be informative, using sample concentration prior to testing relative to replicate testing of samples without pre-processing, in order to gain insight into the potential effect of interfering substances that could be concentrated along with the virus and affect target recovery.

In conclusion, automated replicate testing using the Aptima HIV Quant assay provides a scalable alternative to manual single-copy assays for detecting residual viremia in HIV-infected, ART-suppressed individuals, offering high clinical sensitivity, precision, and reproducibility. With many ongoing cure-directed clinical trials (9, 70, 71), the wide dynamic range and high throughput of the replicate testing-based single-copy assay will be advantageous for measuring the *in vivo* burden of HIV reservoirs.

## SUPPLEMENTAL MATERIAL

Supplemental figures and materials jcm.00995-25-s0001.docx Figures S1 to S3 and Supplemental materials.

ASM does not own the copyrights to Supplemental Material that may be linked to, or accessed through, an article. The authors have granted ASM a non-exclusive, world-wide license to publish the Supplemental Material files. Please contact the corresponding author directly for reuse.

## References

[1] Dornadula G, Zhang H, VanUitert B, Stern J, Livornese L Jr, Ingerman MJ, Witek J, Kedanis RJ, Natkin J, DeSimone J, Pomerantz RJ. 1999. Residual HIV-1 RNA in blood plasma of patients taking suppressive highly active antiretroviral therapy. JAMA 282:1627–1632. doi:10.1001/jama.282.17.1627 10553788

[2] Palmer S, Wiegand AP, Maldarelli F, Bazmi H, Mican JM, Polis M, Dewar RL, Planta A, Liu S, Metcalf JA, Mellors JW, Coffin JM. 2003. New real-time reverse transcriptase-initiated PCR assay with single-copy sensitivity for human immunodeficiency virus type 1 RNA in plasma. J Clin Microbiol 41:4531–4536. doi:10.1128/JCM.41.10.4531-4536.2003 14532178 PMC254331

[3] Bailey JR, Sedaghat AR, Kieffer T, Brennan T, Lee PK, Wind-Rotolo M, Haggerty CM, Kamireddi AR, Liu Y, Lee J, Persaud D, Gallant JE, Cofrancesco J Jr, Quinn TC, Wilke CO, Ray SC, Siliciano JD, Nettles RE, Siliciano RF. 2006. Residual human immunodeficiency virus type 1 viremia in some patients on antiretroviral therapy is dominated by a small number of invariant clones rarely found in circulating CD4+ T cells. J Virol 80:6441–6457. doi:10.1128/JVI.00591-06 16775332 PMC1488985

[4] Anderson JA, Archin NM, Ince W, Parker D, Wiegand A, Coffin JM, Kuruc J, Eron J, Swanstrom R, Margolis DM. 2011. Clonal sequences recovered from plasma from patients with residual HIV-1 viremia and on intensified antiretroviral therapy are identical to replicating viral RNAs recovered from circulating resting CD4+ T cells. J Virol 85:5220–5223. doi:10.1128/JVI.00284-11 21367910 PMC3126162

[5] Simonetti FR, Sobolewski MD, Fyne E, Shao W, Spindler J, Hattori J, Anderson EM, Watters SA, Hill S, Wu X, et al.. 2016. Clonally expanded CD4+ T cells can produce infectious HIV-1 in vivo. Proc Natl Acad Sci USA 113:1883–1888. doi:10.1073/pnas.1522675113 26858442 PMC4763755

[6] White JA, Wu F, Yasin S, Moskovljevic M, Varriale J, Dragoni F, Camilo-Contreras A, Duan J, Zheng MY, Tadzong NF, et al.. 2023. Clonally expanded HIV-1 proviruses with 5’-leader defects can give rise to nonsuppressible residual viremia. J Clin Invest 133:e165245. doi:10.1172/JCI165245 36602866 PMC10014112

[7] Mohammadi A, Etemad B, Zhang X, Li Y, Bedwell GJ, Sharaf R, Kittilson A, Melberg M, Crain CR, Traunbauer AK, et al.. 2023. Viral and host mediators of non-suppressible HIV-1 viremia. Nat Med 29:3212–3223. doi:10.1038/s41591-023-02611-1 37957382 PMC10719098

[8] Deeks SG, Archin N, Cannon P, Collins S, Jones RB, de Jong MAWP, Lambotte O, Lamplough R, Ndung’u T, Sugarman J, Tiemessen CT, Vandekerckhove L, Lewin SR, International AIDS Society (IAS) Global Scientific Strategy working group. 2021. Research priorities for an HIV cure: international AIDS society global scientific strategy 2021. Nat Med 27:2085–2098. doi:10.1038/s41591-021-01590-5 34848888

[9] Abdel-Mohsen M, Richman D, Siliciano RF, Nussenzweig MC, Howell BJ, Martinez-Picado J, Chomont N, Bar KJ, Yu XG, Lichterfeld M, et al.. 2020. Recommendations for measuring HIV reservoir size in cure-directed clinical trials. Nat Med 26:1339–1350. doi:10.1038/s41591-020-1022-1 32895573 PMC7703694

[10] Li JZ, Melberg M, Kittilson A, Abdel-Mohsen M, Li Y, Aga E, Bosch RJ, Wonderlich ER, Kinslow J, Giron LB, et al.. 2024. Predictors of HIV rebound differ by timing of antiretroviral therapy initiation. JCI Insight 9:e173864. doi:10.1172/jci.insight.173864 38329130 PMC10967395

[11] Rosenbloom DIS, Bacchetti P, Stone M, Deng X, Bosch RJ, Richman DD, Siliciano JD, Mellors JW, Deeks SG, Ptak RG, Hoh R, Keating SM, Dimapasoc M, Massanella M, Lai J, Sobolewski MD, Kulpa DA, Busch MP, Reservoir Assay Validation and Evaluation Network (RAVEN) Study Group. 2019. Assessing intra-lab precision and inter-lab repeatability of outgrowth assays of HIV-1 latent reservoir size. PLoS Comput Biol 15:e1006849. doi:10.1371/journal.pcbi.1006849 30978183 PMC6481870

[12] Wonderlich ER, Subramanian K, Cox B, Wiegand A, Lackman-Smith C, Bale MJ, Stone M, Hoh R, Kearney MF, Maldarelli F, Deeks SG, Busch MP, Ptak RG, Kulpa DA. 2019. Effector memory differentiation increases detection of replication-competent HIV-l in resting CD4+ T cells from virally suppressed individuals. PLoS Pathog 15:e1008074. doi:10.1371/journal.ppat.1008074 31609991 PMC6812841

[13] Stone M, Rosenbloom DIS, Bacchetti P, Deng X, Dimapasoc M, Keating S, Bakkour S, Richman DD, Mellors JW, Deeks SG, Lai J, Beg S, Siliciano JD, Pagliuzza A, Chomont N, Lackman-Smith C, Ptak RG, Busch MP. 2021. Assessing the suitability of next-generation viral outgrowth assays to measure human immunodeficiency virus 1 latent reservoir size. J Infect Dis 224:1209–1218. doi:10.1093/infdis/jiaa089 32147687 PMC8514180

[14] Kuzmichev YV, Lackman-Smith C, Bakkour S, Wiegand A, Bale MJ, Musick A, Bernstein W, Aronson N, Ake J, Tovanabutra S, Stone M, Ptak RG, Kearney MF, Busch MP, Wonderlich ER, Kulpa DA. 2023. Application of ultrasensitive digital ELISA for p24 enables improved evaluation of HIV-1 reservoir diversity and growth kinetics in viral outgrowth assays. Sci Rep 13:10958. doi:10.1038/s41598-023-37223-9 37414788 PMC10326067

[15] Levy CN, Hughes SM, Roychoudhury P, Reeves DB, Amstuz C, Zhu H, Huang M-L, Wei Y, Bull ME, Cassidy NAJ, McClure J, Frenkel LM, Stone M, Bakkour S, Wonderlich ER, Busch MP, Deeks SG, Schiffer JT, Coombs RW, Lehman DA, Jerome KR, Hladik F. 2021. A highly multiplexed droplet digital PCR assay to measure the intact HIV-1 proviral reservoir. Cell Rep Med 2:100243. doi:10.1016/j.xcrm.2021.100243 33948574 PMC8080125

[16] Bakkour S, Deng X, Bacchetti P, Grebe E, Montalvo L, Worlock A, Stone M, Deeks SG, Richman DD, Busch MP. 2020. Replicate aptima assay for quantifying residual plasma viremia in individuals on antiretroviral therapy. J Clin Microbiol 58:e01400-20. doi:10.1128/JCM.01400-20 PMC7685884 32967900

[17] Nair M, Gettins L, Fuller M, Kirtley S, Hemelaar J. 2024. Global and regional genetic diversity of HIV-1 in 2010-21: systematic review and analysis of prevalence. Lancet Microbe 5:100912. doi:10.1016/S2666-5247(24)00151-4 39278231

[18] Somsouk M, Dunham RM, Cohen M, Albright R, Abdel-Mohsen M, Liegler T, Lifson J, Piatak M, Gorelick R, Huang Y, Wu Y, Hsue PY, Martin JN, Deeks SG, McCune JM, Hunt PW. 2014. The immunologic effects of mesalamine in treated HIV-infected individuals with incomplete CD4+ T cell recovery: a randomized crossover trial. PLoS One 9:e116306. doi:10.1371/journal.pone.0116306 25545673 PMC4283685

[19] Cillo AR, Vagratian D, Bedison MA, Anderson EM, Kearney MF, Fyne E, Koontz D, Coffin JM, Piatak M, Mellors JW. 2014. Improved single-copy assays for quantification of persistent HIV-1 viremia in patients on suppressive antiretroviral therapy. J Clin Microbiol 52:3944–3951. doi:10.1128/JCM.02060-14 25187636 PMC4313209

[20] Tosiano MA, Jacobs JL, Shutt KA, Cyktor JC, Mellors JW. 2019. A simpler and more sensitive single-copy HIV-1 RNA assay for quantification of persistent HIV-1 viremia in individuals on suppressive antiretroviral therapy. J Clin Microbiol 57:e01714-18. doi:10.1128/JCM.01714-18 30626659 PMC6425167

[21] Cline AN, Bess JW, Piatak M Jr, Lifson JD. 2005. Highly sensitive SIV plasma viral load assay: practical considerations, realistic performance expectations, and application to reverse engineering of vaccines for AIDS. J of Medical Primatology 34:303–312. doi:10.1111/j.1600-0684.2005.00128.x 16128925

[22] Jacobs JL, Tosiano MA, Koontz DL, Staines B, Worlock A, Harrington K, Bakkour S, Stone M, Shutt K, Busch MP, Mellors JW. 2020. Automated multireplicate quantification of persistent HIV-1 viremia in individuals on antiretroviral therapy. J Clin Microbiol 58:e01442-20. doi:10.1128/JCM.01442-20 32967899 PMC7685899

[23] Wu G, Swanson M, Talla A, Graham D, Strizki J, Gorman D, Barnard RJO, Blair W, Søgaard OS, Tolstrup M, Østergaard L, Rasmussen TA, Sekaly R-P, Archin NM, Margolis DM, Hazuda DJ, Howell BJ. 2017. HDAC inhibition induces HIV-1 protein and enables immune-based clearance following latency reversal. JCI Insight 2:e92901. doi:10.1172/jci.insight.92901 28814661 PMC5621903

[24] Stengelin M, Roy D, Aghvanyan A, Kenten J, Sigal GB, Glezer EN, Wohlstadter JN. 2015. HIV p24 immunoassay with the sensitivity of PCR methods. Abstr American association for clinical chemistry (AACC) annual meeting and clinical lab Expo, abstr B-069.

[25] Gandhi RT, Zheng L, Bosch RJ, Chan ES, Margolis DM, Read S, Kallungal B, Palmer S, Medvik K, Lederman MM, Alatrakchi N, Jacobson JM, Wiegand A, Kearney M, Coffin JM, Mellors JW, Eron JJ, AIDS Clinical Trials Group A5244 team. 2010. The effect of raltegravir intensification on low-level residual viremia in HIV-infected patients on antiretroviral therapy: a randomized controlled trial. PLoS Med 7:e1000321. doi:10.1371/journal.pmed.1000321 20711481 PMC2919424

[26] Rasmussen TA, Rajdev L, Rhodes A, Dantanarayana A, Tennakoon S, Chea S, Spelman T, Lensing S, Rutishauser R, Bakkour S, Busch M, Siliciano JD, Siliciano RF, Einstein MH, Dittmer DP, Chiao E, Deeks SG, Durand C, Lewin SR. 2021. Impact of anti-PD-1 and anti-CTLA-4 on the human immunodeficiency virus (HIV) reservoir in people living with HIV with cancer on antiretroviral therapy: the AIDS malignancy consortium 095 study. Clin Infect Dis 73:e1973-81. doi:10.1093/cid/ciaa1530 33677480 PMC8492152

[27] Uldrick TS, Adams SV, Fromentin R, Roche M, Fling SP, Gonçalves PH, Lurain K, Ramaswami R, Wang C-CJ, Gorelick RJ, Welker JL, O’Donoghue L, Choudhary H, Lifson JD, Rasmussen TA, Rhodes A, Tumpach C, Yarchoan R, Maldarelli F, Cheever MA, Sékaly R, Chomont N, Deeks SG, Lewin SR. 2022. Pembrolizumab induces HIV latency reversal in people living with HIV and cancer on antiretroviral therapy. Sci Transl Med 14:eabl3836. doi:10.1126/scitranslmed.abl3836 35080914 PMC9014398

[28] Marzinke MA, Grinsztejn B, Fogel JM, Piwowar-Manning E, Li M, Weng L, McCauley M, Cummings V, Ahmed S, Haines CD, et al.. 2021. Characterization of human immunodeficiency virus (HIV) infection in cisgender men and transgender women who have sex with men receiving injectable cabotegravir for HIV prevention: HPTN 083. J Infect Dis 224:1581–1592. doi:10.1093/infdis/jiab152 33740057 PMC8599849

[29] Koss CA, Gandhi M, Halvas EK, Okochi H, Chu C, Glidden DV, Georgetti Gomez L, Heaps AL, Conroy AA, Tran M, Shetler C, Hoeth D, Kuncze K, Louie A, Rivera Garza H, Wafula Mugoma E, Penrose KJ, Chohan BH, Ayieko JO, Mills A, Patel RR, Mellors JW, Parikh UM. 2024. First case of HIV seroconversion with integrase resistance mutations on long-acting cabotegravir for prevention in routine care. Open Forum Infect Dis 11:ofae468. doi:10.1093/ofid/ofae468 39229286 PMC11370791

[30] Maldarelli F, Palmer S, King MS, Wiegand A, Polis MA, Mican J, Kovacs JA, Davey RT, Rock-Kress D, Dewar R, Liu S, Metcalf JA, Rehm C, Brun SC, Hanna GJ, Kempf DJ, Coffin JM, Mellors JW. 2007. ART suppresses plasma HIV-1 RNA to a stable set point predicted by pretherapy viremia. PLoS Pathog 3:e46. doi:10.1371/journal.ppat.0030046 17411338 PMC1847689

[31] Palmer S, Maldarelli F, Wiegand A, Bernstein B, Hanna GJ, Brun SC, Kempf DJ, Mellors JW, Coffin JM, King MS. 2008. Low-level viremia persists for at least 7 years in patients on suppressive antiretroviral therapy. Proc Natl Acad Sci USA 105:3879–3884. doi:10.1073/pnas.0800050105 18332425 PMC2268833

[32] Dinoso JB, Kim SY, Wiegand AM, Palmer SE, Gange SJ, Cranmer L, O’Shea A, Callender M, Spivak A, Brennan T, Kearney MF, Proschan MA, Mican JM, Rehm CA, Coffin JM, Mellors JW, Siliciano RF, Maldarelli F. 2009. Treatment intensification does not reduce residual HIV-1 viremia in patients on highly active antiretroviral therapy. Proc Natl Acad Sci USA 106:9403–9408. doi:10.1073/pnas.0903107106 19470482 PMC2685743

[33] McMahon D, Jones J, Wiegand A, Gange SJ, Kearney M, Palmer S, McNulty S, Metcalf JA, Acosta E, Rehm C, Coffin JM, Mellors JW, Maldarelli F. 2010. Short-course raltegravir intensification does not reduce persistent low-level viremia in patients with HIV-1 suppression during receipt of combination antiretroviral therapy. Clin Infect Dis 50:912–919. doi:10.1086/650749 20156060 PMC2897152

[34] Swanstrom AE, Gorelick RJ, Welker JL, Schmidt F, Lu B, Wang K, Rowe W, Breed MW, Killoran KE, Kramer JA, Donohue D, Roser JD, Bieniasz PD, Hatziioannou T, Pyle C, Thomas JA, Trubey CM, Zheng J, Blair W, Yant SR, Lifson JD, Del Prete GQ. 2023. Long-acting lenacapavir protects macaques against intravenous challenge with simian-tropic HIV. EBioMedicine 95:104764. doi:10.1016/j.ebiom.2023.104764 37625266 PMC10470178

[35] Mystakelis HA, Wilson E, Laidlaw E, Poole A, Krishnan S, Rupert A, Welker JL, Gorelick RJ, Lisco A, Manion M, Baker JV, Migueles SA, Sereti I. 2023. An open label randomized controlled trial of atorvastatin versus aspirin in elite controllers and antiretroviral-treated people with HIV. AIDS 37:1827–1835. doi:10.1097/QAD.0000000000003656 37450602 PMC10481929

[36] Gay CL, James KS, Tuyishime M, Falcinelli SD, Joseph SB, Moeser MJ, Allard B, Kirchherr JL, Clohosey M, Raines SLM, Montefiori DC, Shen X, Gorelick RJ, Gama L, McDermott AB, Koup RA, Mascola JR, Floris-Moore M, Kuruc JD, Ferrari G, Eron JJ, Archin NM, Margolis DM. 2022. Stable latent HIV infection and low-level viremia despite treatment with the broadly neutralizing antibody VRC07-523LS and the latency reversal agent vorinostat. J Infect Dis 225:856–861. doi:10.1093/infdis/jiab487 34562096 PMC8889279

[37] Gatechompol S, Zheng L, Bao Y, Avihingsanon A, Kerr SJ, Kumarasamy N, Hakim JG, Maldarelli F, Gorelick RJ, Welker JL, Lifson JD, Hosseinipour MC, Eron JJ, Ruxrungtham K. 2021. Prevalence and risk of residual viremia after ART in low- and middle-income countries: a cross-sectional study. Medicine (Baltimore) 100:e26817. doi:10.1097/MD.0000000000026817 34477118 PMC8415996

[38] Kroon EDMB, Ananworanich J, Pagliuzza A, Rhodes A, Phanuphak N, Trautmann L, Mitchell JL, Chintanaphol M, Intasan J, Pinyakorn S, et al.. 2020. A randomized trial of vorinostat with treatment interruption after initiating antiretroviral therapy during acute HIV-1 infection. J Virus Erad 6:100004. doi:10.1016/j.jve.2020.100004 33251022 PMC7646672

[39] Gay CL, Kuruc JD, Falcinelli SD, Warren JA, Reifeis SA, Kirchherr JL, James KS, Dewey MG, Helms A, Allard B, Stuelke E, Gamble A, Plachco A, Gorelick RJ, Eron JJ, Hudgens M, Garrido C, Goonetilleke N, DeBenedette MA, Tcherepanova IY, Nicolette CA, Archin NM, Margolis DM. 2020. Assessing the impact of AGS-004, a dendritic cell-based immunotherapy, and vorinostat on persistent HIV-1 Infection. Sci Rep 10:5134. doi:10.1038/s41598-020-61878-3 32198428 PMC7083965

[40] Reid EG, Suazo A, Lensing SY, Dittmer DP, Ambinder RF, Maldarelli F, Gorelick RJ, Aboulafia D, Mitsuyasu R, Dickson MA, Wachsman W, AIDS Malignancy Consortium (AMC). 2020. Pilot trial AMC-063: safety and efficacy of bortezomib in AIDS-associated kaposi sarcoma. Clin Cancer Res 26:558–565. doi:10.1158/1078-0432.CCR-19-1044 31624104 PMC7034393

[41] Boulougoura A, Gabriel E, Laidlaw E, Khetani V, Arakawa K, Higgins J, Rupert A, Gorelick RJ, Lumbard K, Pau A, Poole A, Kibiy A, Kumar P, Sereti I. 2019. A phase I, randomized, controlled clinical study of CC-11050 in people living with HIV with suppressed plasma viremia on antiretroviral therapy (APHRODITE). Open Forum Infect Dis 6:ofz246. doi:10.1093/ofid/ofz246 31211164 PMC6559277

[42] Crowell TA, Colby DJ, Pinyakorn S, Sacdalan C, Pagliuzza A, Intasan J, Benjapornpong K, Tangnaree K, Chomchey N, Kroon E, et al.. 2019. Safety and efficacy of VRC01 broadly neutralising antibodies in adults with acutely treated HIV (RV397): a phase 2, randomised, double-blind, placebo-controlled trial. Lancet HIV 6:e297–e306. doi:10.1016/S2352-3018(19)30053-0 31000477 PMC6693657

[43] Scully EP, Gandhi M, Johnston R, Hoh R, Lockhart A, Dobrowolski C, Pagliuzza A, Milush JM, Baker CA, Girling V, Ellefson A, Gorelick R, Lifson J, Altfeld M, Alter G, Cedars M, Solomon A, Lewin SR, Karn J, Chomont N, Bacchetti P, Deeks SG. 2019. Sex-based differences in human immunodeficiency virus type 1 reservoir activity and residual immune activation. J Infect Dis 219:1084–1094. doi:10.1093/infdis/jiy617 30371873 PMC6784502

[44] Lee SA, Elliott JH, McMahon J, Hartogenesis W, Bumpus NN, Lifson JD, Gorelick RJ, Bacchetti P, Deeks SG, Lewin SR, Savic RM. 2019. Population pharmacokinetics and pharmacodynamics of disulfiram on inducing latent HIV‐1 transcription in a phase IIb trial. Clin Pharmacol Ther 105:692–702. doi:10.1002/cpt.1220 30137649 PMC6379104

[45] Colby DJ, Trautmann L, Pinyakorn S, Leyre L, Pagliuzza A, Kroon E, Rolland M, Takata H, Buranapraditkun S, Intasan J, et al.. 2018. Rapid HIV RNA rebound after antiretroviral treatment interruption in persons durably suppressed in Fiebig I acute HIV infection. Nat Med 24:923–926. doi:10.1038/s41591-018-0026-6 29892063 PMC6092240

[46] Lynch RM, Boritz E, Coates EE, DeZure A, Madden P, Costner P, Enama ME, Plummer S, Holman L, Hendel CS, et al.. 2015. Virologic effects of broadly neutralizing antibody VRC01 administration during chronic HIV-1 infection. Sci Transl Med 7:319ra206. doi:10.1126/scitranslmed.aad5752 PMC12366723 26702094

[47] Elliott JH, McMahon JH, Chang CC, Lee SA, Hartogensis W, Bumpus N, Savic R, Roney J, Hoh R, Solomon A, Piatak M, Gorelick RJ, Lifson J, Bacchetti P, Deeks SG, Lewin SR. 2015. Short-term administration of disulfiram for reversal of latent HIV infection: a phase 2 dose-escalation study. Lancet HIV 2:e520–9. doi:10.1016/S2352-3018(15)00226-X 26614966 PMC5108570

[48] Caskey M, Klein F, Lorenzi JCC, Seaman MS, West AP, Buckley N, Kremer G, Nogueira L, Braunschweig M, Scheid JF, Horwitz JA, Shimeliovich I, Ben-Avraham S, Witmer-Pack M, Platten M, Lehmann C, Burke LA, Hawthorne T, Gorelick RJ, Walker BD, Keler T, Gulick RM, Fätkenheuer G, Schlesinger SJ, Nussenzweig MC. 2015. Viraemia suppressed in HIV-1-infected humans by broadly neutralizing antibody 3BNC117. Nature 522:487–491. doi:10.1038/nature14411 25855300 PMC4890714

[49] Riddler SA, Zheng L, Durand CM, Ritz J, Koup RA, Ledgerwood J, Bailer RT, Koletar SL, Eron JJ, Keefer MC, Macatangay BJC, Cyktor JC, Mellors JW, AIDS Clinical Trials Group A5342 Protocol Team. 2018. Randomized clinical trial to assess the impact of the broadly neutralizing HIV-1 monoclonal antibody VRC01 on HIV-1 persistence in individuals on effective ART. Open Forum Infect Dis 5:ofy242. doi:10.1093/ofid/ofy242 30364428 PMC6195652

[50] Cyktor JC, Bosch RJ, Mar H, Macatangay BJ, Collier AC, Hogg E, Godfrey C, Eron JJ, McMahon DK, Mellors JW, Gandhi RT, ACTG A5321 Team. 2021. Association of male sex and obesity with residual plasma human immunodeficiency virus 1 viremia in persons on long-term antiretroviral therapy. J Infect Dis 223:462–470. doi:10.1093/infdis/jiaa373 32603416 PMC7881329

[51] Henrich TJ, Bosch RJ, Godfrey C, Mar H, Nair A, Keefer M, Fichtenbaum C, Moisi D, Clagett B, Buck AM, et al.. 2024. Sirolimus reduces T cell cycling, immune checkpoint marker expression, and HIV-1 DNA in people with HIV. Cell Rep Med 5:101745. doi:10.1016/j.xcrm.2024.101745 39321793 PMC11513808

[52] Hsu J, Van Besien K, Glesby MJ, Pahwa S, Coletti A, Warshaw MG, Petz L, Moore TB, Chen YH, Pallikkuth S, Dhummakupt A, Cortado R, Golner A, Bone F, Baldo M, Riches M, Mellors JW, Tobin NH, Browning R, Persaud D, Bryson Y, International Maternal Pediatric Adolescent AIDS Clinical Trials Network (IMPAACT) P1107 Team. 2023. HIV-1 remission and possible cure in a woman after haplo-cord blood transplant. Cell 186:1115–1126. doi:10.1016/j.cell.2023.02.030 36931242 PMC10616809

[53] Eshleman SH, Fogel JM, Piwowar-Manning E, Chau G, Cummings V, Agyei Y, Richardson P, Sullivan P, Haines CD, Bushman LR, et al.. 2022. Characterization of human immunodeficiency virus (HIV) infections in women who received injectable cabotegravir or tenofovir disoproxil fumarate/emtricitabine for HIV prevention: HPTN 084. J Infect Dis 225:1741–1749. doi:10.1093/infdis/jiab576 35301540 PMC9113509

[54] Yukl SA, Li P, Fujimoto K, Lampiris H, Lu CM, Hare CB, Deeks SG, Liegler T, Pandori M, Havlir DV, Wong JK. 2011. Modification of the Abbott RealTime assay for detection of HIV-1 plasma RNA viral loads less than one copy per milliliter. J Virol Methods 175:261–265. doi:10.1016/j.jviromet.2011.04.015 21536073 PMC3827908

[55] Gupta RK, Abdul-Jawad S, McCoy LE, Mok HP, Peppa D, Salgado M, Martinez-Picado J, Nijhuis M, Wensing AMJ, Lee H, Grant P, Nastouli E, Lambert J, Pace M, Salasc F, Monit C, Innes AJ, Muir L, Waters L, Frater J, Lever AML, Edwards SG, Gabriel IH, Olavarria E. 2019. HIV-1 remission following CCR5Δ32/Δ32 haematopoietic stem-cell transplantation. Nature 568:244–248. doi:10.1038/s41586-019-1027-4 30836379 PMC7275870

[56] Hatano H, Yukl SA, Ferre AL, Graf EH, Somsouk M, Sinclair E, Abdel-Mohsen M, Liegler T, Harvill K, Hoh R, Palmer S, Bacchetti P, Hunt PW, Martin JN, McCune JM, Tracy RP, Busch MP, O’Doherty U, Shacklett BL, Wong JK, Deeks SG. 2013. Prospective antiretroviral treatment of asymptomatic, HIV-1 infected controllers. PLoS Pathog 9:e1003691. doi:10.1371/journal.ppat.1003691 24130489 PMC3795031

[57] Landay A, Golub ET, Desai S, Zhang J, Winkelman V, Anastos K, Durkin H, Young M, Villacres MC, Greenblatt RM, Norris PJ, Busch MP, Womenʼs Interagency HIV Study. 2014. HIV RNA levels in plasma and cervical-vaginal lavage fluid in elite controllers and HAART recipients. AIDS 28:739–743. doi:10.1097/QAD.0000000000000150 24326356 PMC4160049

[58] Chang L, Song L, Fournier DR, Kan CW, Patel PP, Ferrell EP, Pink BA, Minnehan KA, Hanlon DW, Duffy DC, Wilson DH. 2013. Simple diffusion-constrained immunoassay for p24 protein with the sensitivity of nucleic acid amplification for detecting acute HIV infection. J Virol Methods 188:153–160. doi:10.1016/j.jviromet.2012.08.017 23036750

[59] Cabrera C, Chang L, Stone M, Busch M, Wilson DH. 2015. Rapid, fully automated digital immunoassay for p24 protein with the sensitivity of nucleic acid amplification for detecting acute HIV Infection. Clin Chem 61:1372–1380. doi:10.1373/clinchem.2015.243287 26369787

[60] Wilson DH, Rissin DM, Kan CW, Fournier DR, Piech T, Campbell TG, Meyer RE, Fishburn MW, Cabrera C, Patel PP, Frew E, Chen Y, Chang L, Ferrell EP, von Einem V, McGuigan W, Reinhardt M, Sayer H, Vielsack C, Duffy DC. 2016. The Simoa HD-1 analyzer: a novel fully automated digital immunoassay analyzer with single-molecule sensitivity and multiplexing. SLAS Technol 21:533–547. doi:10.1177/2211068215589580 26077162

[61] Stone M, Bainbridge J, Sanchez AM, Keating SM, Pappas A, Rountree W, Todd C, Bakkour S, Manak M, Peel SA, Coombs RW, Ramos EM, Shriver MK, Contestable P, Nair SV, Wilson DH, Stengelin M, Murphy G, Hewlett I, Denny TN, Busch MP. 2018. Comparison of detection limits of fourth- and fifth-generation combination HIV antigen-antibody, p24 antigen, and viral load assays on diverse HIV isolates. J Clin Microbiol 56:e02045–17. 29793968 10.1128/JCM.02045-17 PMC6062817

[62] Passaes CPB, Bruel T, Decalf J, David A, Angin M, Monceaux V, Muller-Trutwin M, Noel N, Bourdic K, Lambotte O, Albert ML, Duffy D, Schwartz O, Sáez-Cirión A. 2017. Ultrasensitive HIV-1 p24 assay detects single infected cells and differences in reservoir induction by latency reversal agents. J Virol 91:e02296-16. doi:10.1128/JVI.02296-16 28077644 PMC5331803

[63] Stuelke EL, James KS, Kirchherr JL, Allard B, Baker C, Kuruc JD, Gay CL, Margolis DM, Archin NM. 2020. Measuring the inducible, replication-competent HIV reservoir using an ultra-sensitive p24 readout, the digital ELISA viral outgrowth assay. Front Immunol 11:1971. doi:10.3389/fimmu.2020.01971 32849659 PMC7423995

[64] Levinger C, Howard JN, Cheng J, Tang P, Joshi A, Catalfamo M, Bosque A. 2021. An ultrasensitive planar array p24 Gag ELISA to detect HIV-1 in diverse biological matrixes. Sci Rep 11:23682. doi:10.1038/s41598-021-03072-7 34880361 PMC8654962

[65] Wu G, Cheney C, Huang Q, Hazuda DJ, Howell BJ, Zuck P. 2021. Improved detection of HIV gag p24 protein using a combined immunoprecipitation and digital ELISA method. Front Microbiol 12:636703. doi:10.3389/fmicb.2021.636703 33796087 PMC8007784

[66] Falcinelli SD, Peterson JJ, Turner A-MW, Irlbeck D, Read J, Raines SL, James KS, Sutton C, Sanchez A, Emery A, et al.. 2022. Combined noncanonical NF-κB agonism and targeted BET bromodomain inhibition reverse HIV latency ex vivo. J Clin Invest 132:e157281. doi:10.1172/JCI157281 35426377 PMC9012286

[67] Wietgrefe SW, Anderson J, Duan L, Southern PJ, Zuck P, Wu G, Howell BJ, Reilly C, Kroon E, Chottanapund S, et al.. 2023. Initial productive and latent HIV infections originate in vivo by infection of resting T cells. J Clin Invest 133:e171501. doi:10.1172/JCI171501 37733443 PMC10645380

[68] Wirden M, Palich R, Abdi B, Valantin MA, Tubiana R, Schneider L, Seang S, Faycal A, Sellem B, Katlama C, Calvez V, Marcelin AG. 2022. More HIV-1 RNA detected and quantified with the Cobas 6800 system in patients on antiretroviral therapy. J Antimicrob Chemother 77:2251–2256. doi:10.1093/jac/dkac174 35640662

[69] Henrich TJ, Schreiner C, Cameron C, Hogan LE, Richardson B, Rutishauser RL, Deitchman AN, Chu S, Rogers R, Thanh C, Gibson EA, Zarinsefat A, Bakkour S, Aweeka F, Busch MP, Liegler T, Baker C, Milush J, Deeks SG, Stock PG. 2021. Everolimus, an mTORC1/2 inhibitor, in ART-suppressed individuals who received solid organ transplantation: a prospective study. Am J Transplant 21:1765–1779. doi:10.1111/ajt.16244 32780519 PMC9177122

[70] Ta TM, Malik S, Anderson EM, Jones AD, Perchik J, Freylikh M, Sardo L, Klase ZA, Izumi T. 2022. Insights into persistent HIV-1 infection and functional cure: novel capabilities and strategies. Front Microbiol 13:862270. doi:10.3389/fmicb.2022.862270 35572626 PMC9093714

[71] Armani-Tourret M, Bone B, Tan TS, Sun W, Bellefroid M, Struyve T, Louella M, Yu XG, Lichterfeld M. 2024. Immune targeting of HIV-1 reservoir cells: a path to elimination strategies and cure. Nat Rev Microbiol 22:328–344. doi:10.1038/s41579-024-01010-8 38337034 PMC11131351

