# Supplementary material for: Comparison of manual and automated ultrasensitive assays for residual HIV-1 in plasma from individuals on suppressive antiretroviral therapy
Source: J Clin Microbiol. 2025 Nov 18;63(12):e00995-25. doi: 10.1128/jcm.00995-25 (PMC12710318; doi:10.1128/jcm.00995-25)
Supplement: Supplemental figures and materials — Figures S1 to S3 and Supplemental materials. [file jcm.00995-25-s0001.docx]

**Supplemental Figure 1: Measured HIV p24 using ultrasensitive immunoassays in qualification phase plasma panels.** Left panel: For each HIV subtype (B and C), plasma from two different individuals, identified as HIV RNA-positive and Ab-negative through routine blood donor testing, was serially diluted, blinded, and each concentration was included in duplicate in the panel for measurement of HIV p24 using ultrasensitive assays. Middle panel: For each HIV subtype (B and C), plasma samples from individuals identified as HIV RNA-negative and Ab-positive through routine blood donor testing were blinded and included in singlicate in qualification phase panels for testing using ultrasensitive HIV p24 assays. Right panel: Plasma samples from 10 HIV-negative individuals were included in singlicate in each of the two HIV subtype-specific qualification panels.

**Supplemental Figure 2: Measured VL in plasma samples from HIV-positive RAVEN participants not on antiviral therapy.** Each sample was blinded and included in duplicate in the Evaluation Phase panel for testing using single copy assays.


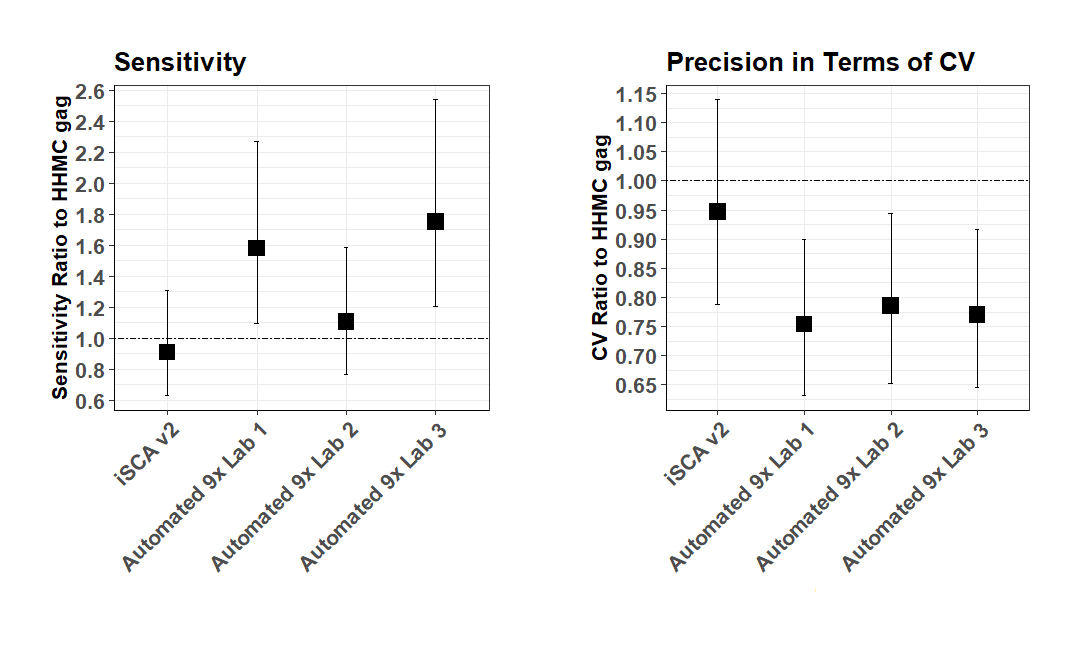


**Supplemental Figure 3: Relative sensitivity and precision of single copy assays used to test the Evaluation Phase panel.** Performance of each assay was modeled using statistical analysis relative to the HMMC gag assay as a reference. Error bars around the point estimate depict the 95% confidence interval.

**SUPPLEMENTAL MATERIALS**

**Statistical Analysis of Sensitivity and Precision**

We compared the assays’ performance using statistical models of their sensitivity and precision. We modeled the probability of a positive result assuming that the number of copies in each sample assayed followed a Poisson distribution and that each copy had an independent probability of detection equal to *S_a_* for each assay *a*. The true mean number of copies expected in a sample to be assayed is unknown; we denote it by λ_j_*v_ajk_*, where λ_j_ is the true copies per mL in the *j*th source (donor or diluted stock) that the sample was aliquoted from and *v_ajk_* is the volume in mL of the *k*th aliquot from the *j*th source measured by assay *a*. (For dilution series, we set *v_ajk_* to be the nominal volume for the aliquots that targeted 1 copy/mL, with the other dilutions having *v_ajk_* equal to the nominal volume times the target copies per mL.) The probability of a positive result is then:

1 - exp(-λ_j_*v_ajk_S_a_*) (1)

This is the Poisson probability of having at least 1 copy present, when the expected number of copies λ_j_*v_ajk_* has been reduced by the factor *S_a_* so that it is now the expected number of detected copies. Note that the λ_j_ and *S_a_* are not separately identifiable; for example, multiplying all the λ_j_ by 2 and dividing all the *S_a_* by 2 will leave all probabilities of a positive result unchanged. We therefore designated the HHMC gag assay as a reference (which we denote with index *a*=0) and estimated the sensitivity of the other assays relative to that of the HHMC gag assay. We included log(*v_ajk_*) as an offset (or “exposure”) variable in order to account for the volume assayed. Our model was:

log(λ_j_*S_a_*) = µ + γ*_j_* + *r_a_* , (2)

where µ is a global intercept term reflecting both *S*_0_ and the λ_j_, the γ*_j_* are random effects that we assume are normally distributed with mean zero and standard deviation σ, and *r_a_* = log(*S_a_*/*S*_0_). We estimated the model’s parameters, µ, σ, and the *r_a_*, by maximum likelihood using the NLmixed procedure in SAS 9.4 (SAS Institute, Cary, NC). Specifically, we found the parameter values that made the fitted probability of the observed pattern of positive and negative assay results the most likely under the model specified by (1) and (2). We report the back-transformed relative sensitivities, exp(*r_a_*) = *S_a_*/*S*_0_, with p-values for the null hypothesis of equal sensitivity, *S_a_*/*S*_0_ = 1.

To compare assays’ precision, we estimated the ratios of their coefficients of variation (CV). The CV is equal to the standard deviation (SD) divided by the mean, so it is a measure of relative precision. Under the constant dispersion formulation of negative binomial regression, the CV for a given assay *a* measuring a given aliquot *k* from a given source *j* is equal to [See: <https://www.stata.com/support/faqs/statistics/nbreg-variance-function/>]:

${CV}_{ajk}=\frac{\sqrt{\left. \mu_{ajk}(1+\delta_{a} \right)}}{\mu_{ajk}}$ (3)

where 𝜇_𝑎𝑗𝑘_ is the expected number of copies for the aliquot and 𝛿_𝑎_ is the dispersion parameter for assay *a*, which is assumed to be the same for all aliquots. We obtained estimates of the ratio of CVs for different assays from a negative binomial regression model with the usual log-linear formulation:

log(𝜇𝑎𝑗𝑘) = µ + γ*_j_* + *f_a_* . (4)

As in equation (2), µ is a global intercept term for the reference assay, which we again set to be the HHMC gag assay, and the γ*_j_* are random effects with mean zero and standard deviation σ. The scale factors *f_a_* allow for the other assays to be consistently higher or lower than HHMC gag on average. We fit the constant-dispersion negative binomial model by maximum likelihood using the NLmixed procedure in SAS 9.4 (SAS Institute, Cary, NC) and then used the parameters to estimate the desired precision comparison on the log scale:

log(CV*_ajk_*/CV_0_*_jk_*) = 0.5*[ log(1+𝛿_𝑎_) - log(1+𝛿_0_) - *f_a_*] (5)

Note that this is the same for all *j* and *k*, because all the terms involving them cancel out. We then exponentiated this quantity to obtain the desired estimate of relative precision. We report the back-transformed relative CV with an exponential function of estimates of the left side in the equation (5), with p-values for the null hypothesis of equal precision, CV*_ajk_*/CV_0_*_jk_* = 1.

**Algorithm for Estimating Viral Load from Replicate Measurements**

This algorithm processes replicate Aptima assay measurements to estimate viral load in copies per mL. It handles non-numeric or low-copy replicates, distinguishes between low and high viral load regimes, and applies appropriate estimation methods accordingly.

1. Preprocessing Non-Numeric Replicates

Replicates with non-numeric or qualitative results are converted to numeric approximations:

- "<30" → 5.5

- "<1" → 0.5

- "<0.3" or "Not Detected" → classified as negative

2. Definitions

- K: Number of replicates with ≥30 copies (indicating strong signal)

- Neg: Number of negative replicates

- n: Total number of replicates

3. Mode Selection and Calculation

A. Poisson Mode (Low Copy Number)

Used when Neg > K (i.e., more negative than strong-positive replicates):

- Copy per reaction = −log(Neg / n) × 1.6

- Copy per mL = Copy per reaction × 2

- Confidence Interval (CI):

CI = Copy/mL ± [sqrt((n - Neg) / (n × Neg)) × 1.96 × 3.2]

- If lower CI bound is negative, it is set to 0

- Based on: https://www.ncbi.nlm.nih.gov/pmc/articles/PMC4858304/

B. Average Mode (High Copy Number)

Used when Neg ≤ K (strong signal dominates):

- Negative replicates are removed as inconsistent with high copy assumptions

- Extreme outliers (>3× mean) are removed

- Copy per mL = Arithmetic mean of remaining replicates

- Confidence Interval:

- In R (DescTools): MeanCI(reps, conf.level = 0.95)
